# National and subnational variations in gender relations and the utilization of maternal healthcare services in Nigeria

**DOI:** 10.11604/pamj.2022.42.28.25689

**Published:** 2022-05-12

**Authors:** Yemi Adewoyin, Clifford Obby Odimegwu, Theresa Bassey, Olukemi Funmilayo Awelewa, Oluwakemi Akintan

**Affiliations:** 1 Department of Geography, University of Nigeria, Nsukka, Nigeria,; 2 Demography and Population Studies Programme Schools of Public Health and Social Sciences University of the Witwatersrand, Johannesburg, South Africa,; 3 Department of Environmental Health, College of Health Technology, Calabar, Nigeria,; 4 Department of Geography, University of Ibadan, Ibadan, Nigeria,; 5 Department of Geography and Planning Science, Ekiti State University, Ado-Ekiti, Nigeria

**Keywords:** Maternal health, antenatal care services, health facility delivery, gender relations, subnational regions, Nigeria

## Abstract

**Introduction:**

poor maternal health outcomes remain a major public health issue in Nigeria. These have been shown to be affected by the low level of utilization of maternal healthcare services. This study investigates the levels of gender relations (GR) among Nigerian women and how these influence their utilization of maternal healthcare services. The relations are conceptualized as feminine (FGR), masculine (MGR) and egalitarian.

**Methods:**

data on household decision-making, antenatal care (ANC) visits, health facility delivery, and associated sociodemographic variables, were extracted from the 2018 Nigeria Demographic and Health Survey for 29,992 parous women aged 15-49 for a cross-sectional study. Associations were investigated using Chi-Square and regression analyses.

**Results:**

women with FGR constituted 5.7% of the population at the national level, while subnational variations ranged from 1.8% in the North-East to 12.8% in the South-South regions. The prevalence rates of the recommended minimum ANC visits (RMANC) and health facility delivery were 42.1% and 30.0% at the national level and were lowest in the northern regions. At both the unadjusted and adjusted levels, FGR was not significantly associated with RMANC and health facility delivery at the national level and in all the regions except the South-West. MGR was however significantly associated with increased odds of RMANC (OR: 2.235, CI: 2.043-2.444) and health facility delivery (OR: 2.571, CI: 2.369-2.791) at national level. Significant subnational variations in the association between gender relations and the utilization of maternal healthcare services were also recorded.

**Conclusion:**

sub-national variations in GR and their varying impacts on the utilization of maternal healthcare services in Nigeria suggest that gender-related policies to improve maternal health outcomes should be location-specific, rather than general. As FGR did not affect maternal healthcare services utilization, educating men on the benefits of supporting their wives to scale-up utilization is recommended.

## Introduction

With an estimated 534 maternal deaths per 100,000 live births in 2017, Sub-Saharan Africa (SSA) bears the highest global burden of maternal mortality according to the World Health Organization [1]. The rate also accounts for 99% of maternal mortality in all developing countries [2]. Of the ten countries with the poorest maternal mortality ratio (MMR) in the world at the dawn of the new millennium, nine were from SSA, with the exception being Afghanistan [3]. The countries were Rwanda (1,160), Nigeria (1,200), Guinea-Bissau (1,210), Somalia (1,210), Central African Republic (1,280), Eritrea (1,280), Chad (1,420), South Sudan (1,730) and Sierra-Leone (2,480). While all the countries have made tremendous progress in reducing their MMR by the year 2017, progress in Nigeria has, however, been the slowest. Although better than Sierra Leone (1,120), South Sudan (1,150) and Chad (1,140) in absolute terms, Nigeria´s 917 deaths per 100,000 live births in 2017, relative to its MMR in the year 2000, is indicative of a poor performance. Given its estimated 200 million population, projected to double by the year 2050 when one in every twenty-five people around the globe, will reside in Nigeria [4], the high prevalence of maternal mortality in Nigeria matters for global sustainable development.

Various arguments have been advanced in studies to explain and address the poor maternal health outcomes in Nigeria. Most of these arguments centre on the utilization of antenatal care (ANC) services by pregnant women, the use of health facilities for pregnancy delivery, and the factors that determine women´s health seeking for both services. This is particularly so as the percentage of pregnant women who had the WHO recommended minimum ANC visits (RMANC) - four visits, had only oscillated around 51% between 2003 and 2013 while health facility delivery accounted for between 33% and 36% of all deliveries in the same period [5]. The commonest determinants identified in the studies are age, marital status, literacy, socioeconomic status, number of children previously born, place of residence, employment status, physical accessibility to the services, quality of services, culture and religion, and media access [6-22]. Having someone to accompany the pregnant woman was also identified as a determinant [7, 23, 24]. Less prominent in the studies on the determinants of ANC services and health facility delivery utilization in Nigeria, in spite of its capacity to influence decision-making on health seeking and health outcomes as shown elsewhere [25-32], is the role of gender relations within the households.

Gender relations within the households are expressed, among other ways, in decision-making powers on aspects of household activities including accessing ANC services during pregnancy and using health facilities for delivery. The decision-making powers could be largely male-dominated (masculine), female-dominated (feminine) or equal between both genders. In Nigeria, gender relations are mostly patriarchal and rooted in culture and religion. With the growing clamour for gender equality and women autonomy in Nigeria, as elsewhere, following the Fourth World Conference on Women in 1995, gender relations within Nigerian households have undergone some changes [33]. Owing, however, to the cultural diversity among ethnic groups in Nigeria and the divergent belief systems in households, pattern of gender relations is not uniform in households and across all ethnic segments. Some cultures are more receptive to change than others. Therefore, findings from the very few studies that referenced the role of gender relations in maternal healthcare utilization in Nigeria without taking into account the subnational variation in gender relations [10, 11, 23, 34-37], become inadequate in informing policies to improve maternal health in different geographical regions of Nigeria with their different levels of health outcomes [13, 37-40].

Given this background, this study investigates national and subnational variations in gender relations in Nigerian households and how these impact the utilization of maternal healthcare services in the households. The overarching hypothesis of study is that increased feminine gender relations do not translate to increased utilization of ANC services and health facility delivery at national and subnational levels in Nigeria. Gender, as a social construct, refers to roles and cultural values ascribed to biological sex and associated activities and responsibilities connected to being male or female [41-43]. Following Sanchez-Lopez and Liminana-Gras [44] and Bravo-Baumann [45], gender relations are the interactions among people based on their gender and how rights and responsibilities are ascribed among men and women in societies. These rights and responsibilities, at household levels, include how decision-making powers are distributed between the male and female heads of the households. For this study therefore, feminine gender relations are conceptualized as more decision-making controls for the female over her male partner / spouse while masculine gender relations refer to more decision-making controls for the male [41-45].

## Methods

### Study setting

The study was situated in Nigeria, a country of about 200 million people and over 380 ethnic groups as identified by the 2013 Nigerian Demographic and Health Survey (NDHS). The country operates a Federal System of government using States as administrative units at subnational level. The States are grouped into six geopolitical regions to reflect cultural background, contiguity and common values among the different ethnic groups. While the North-West, South-East and South-West regions are each largely homogenous in ethnicity and culture, the North-East, North Central and South-South regions are an assemblage of different but related ethnic groups, language, and culture. The regions were employed in this study to depict the subnational variations variables of study.

### Data source and study population

The data source for this study was the 2018 Nigerian Demographic and Health Survey (NDHS). The NDHS is a nationally representative survey that employed multi-stage sampling techniques to collect up-to-date estimates of basic demographic and health indicators of the Nigerian population [33]. The survey sample comprised about 42,000 households selected from 74 urban and rural strata in the 36 States and the Federal Capital Territory of Nigeria. By weighting the samples to balance the distribution across clusters in the two-stage stratified cluster samples, the survey covered a total of 41,821 women between the ages of 15 and 49. Of the weighted 41,821 women, 29,992 were parous and therefore qualified to have used maternal healthcare services. The parous women constituted the study sample.

### Variable definition

Two main outcome variables were employed in this study as corollaries of maternal healthcare services. These are ANC utilization and the use of health facilities for pregnancy delivery. In the women´s Individual Recode file of the NDHS datasets, ANC utilization was captured as ‘number of antenatal visits during pregnancy´ in variable (V) M14$1. Following the WHO recommendation of a minimum of four ANC visits, the numeric responses were recoded as < 4 and 4+. Women who had 4+ ANC visits were therefore considered as having the recommended minimum ANC visits (RMANC). Place of delivery (VM15$1) in the file had nine options for the respondents to choose from. The options were different variants of delivery at home, in a public health facility or a private health facility. The options were recoded to the binary form of health facility delivery and non-health facility delivery. Gender relations were derived from variables 632, 739, 743A, 743B, 743D and 743F. These variables measured the women´s decision-making powers by asking who decided on the respondents´ use of contraceptives, how their earnings were spent, their healthcare, large household purchases, visits to family and relatives, and how their spouses´ earnings are spent. The responses were recoded to show three levels of household decision-making as feminine, masculine and equal. Other sociodemographic confounders employed in the study were age (V13), region of residence (V24), place of residence (V25), education (V106), religion (V130), wealth (V190), number of children ever born (V201), marital status (V502), partner´s education (V701), occupation (V717), media access (derived from V120 and V121), and experience of intimate partner violence (derived from variables D104, D106, D107 and D108). The variables have all been shown to impact the utilization of maternal healthcare services.

### Data analysis

Data analysis was carried out using univariate, bivariate and multivariate statistical techniques. The sociodemographic characteristics of the respondents at both the national and subnational levels were analysed using frequency and percentages, while Chi-Square test was used to investigate the association between the sociodemographic variables and the outcome variables. Binary logistic regression was used to model the predictive capacity of gender relations on ANC utilization and health facility delivery at the national level and in each of the six regions separately at both the unadjusted and adjusted levels while controlling for the sociodemographic confounders. The need to control for the sociodemographic variables was predicated on their capacity to influence the regression outcomes because of their underlying relationship with gender relations in the households. In all, a total of 14 regression models were generated to determine the relationship between the predictors and the utilization of maternal healthcare services. Confidence interval of 95% was set for the acceptance of results of the analyses.

## Results

### Sociodemographic characteristics of study population

The geographical distribution of the 29,992 respondents shows that 18.2%, 19.0%, 25.8% and 12.1% were from the North-Central (NC), North-East (NE), North-West (NW) and South-East (SE) regions of Nigeria respectively while the South-South (SS) and South-West (SW) regions had 11.7% and 13.3% representation respectively. While the pattern of distribution of age, marital status, number of children ever born, experience of intimate partner violence, media exposure, and occupation was uniform across the six regions, the regions had marked differences on other variables. As illustrated in Table 1, there were more urban residents in the SE and SW, Islam was more prevalent in the northern regions, while respondents and their partners with secondary education and from richer households were more prevalent in the south. Although in varying proportions, feminine gender relations were the lowest forms of relations at both the national and regional levels. The proportion of women who had the RMANC was lowest in the NE and NW and the regions´ hospital delivery prevalence was even lower than the 30.0% national average.

**Table 1:** sociodemographic characteristics of study population at national and regional levels

Spatial Unit	National (%)	NC (%)	NE (%)	NW (%)	SE (%)	SS (%)	SW (%)
**Variables**	N=29,992	N=5,452	N=5,694	N=7,745	N=3,617	N=3,501	N=3,983
**Age**							
15-19	4.0	3.3	5.8	5.9	2.2	2.8	1.3
20-24	14.4	14.3	18.4	18.2	8.5	11.3	9.6
25-29	20.4	22.4	21.6	21.1	17.7	18.3	18.5
30-34	18.5	17.8	17.5	17.7	19.0	19.7	20.7
35-39	17.2	17.4	15.6	14.5	19.2	18.9	21.1
40-44	13.0	12.5	11.7	11.7	14.5	15.5	14.7
45-49	12.6	12.3	9.5	10.8	18.9	13.5	14.1
**Marital Status**							
Never in Union	3.0	2.0	2.2	0.3	4.6	9.5	3.8
In Union/Living with a man	89.9	90.5	92.4	96.0	83.2	79.8	88.6
Formerly in Union	7.1	7.5	5.3	3.7	12.2	10.7	7.6
**Types of Residence**							
Urban	37.5	31.9	21.1	26.1	63.7	33.6	70.9
Rural	62.5	68.1	78.9	73.9	36.3	66.4	29.1
**Religion**							
Catholic	9.7	14.3	1.3	1.1	41.0	10.0	3.6
Other Christian	36.3	38.5	18.2	4.0	58.1	83.1	61.1
Islam	53.0	46.7	80.4	94.3	0.2	2.3	35.0
Traditional/Others	0.9	0.5	0.0	0.6	0.7	4.6	0.3
**Highest Education**							
No education	41.5	36.7	66.3	74.4	6.3	7.6	10.5
Primary	17.8	21.9	13.9	11.1	25.3	23.0	19.7
Secondary	31.8	30.9	15.9	11.3	55.8	57.5	51.7
Higher	8.8	10.5	3.9	3.2	12.7	12.0	18.1
Partner Highest Education*							
No education	30.3	26.3	55.4	60.2	3.4	3.8	9.7
Primary	14.0	15.5	11.1	11.3	35.5	17.0	14.5
Secondary	30.6	39.3	21.7	16.6	50.6	60.3	49.6
Higher	13.9	18.9	11.8	11.8	10.5	19.0	26.3
**Wealth Status**							
Poor	42.4	38.6	67.9	64.8	16.9	17.4	12.8
Middle	21.2	25.4	17.7	17.8	26.8	26.4	17.2
Rich	36.4	36.0	14.4	17.5	56.3	56.3	70.0
**Children ever born**							
1 - 4	59.7	62.6	54.1	47.8	62.4	70.1	75.3
5 - 8	32.4	33.5	34.8	35.9	34.3	27.1	23.7
9+	7.9	3.9	11.1	16.3	3.2	2.9	1.0
**Experienced Partner Violence**							
Yes	11.2	13.3	12.8	6.4	14.9	14.8	9.1
No	88.8	86.7	87.2	93.6	85.1	85.2	90.9
Access to Radio/TV*							
Yes	70.0	74.1	80.4	60.0	65.6	66.5	81.9
No	28.9	25.9	19.6	40.0	34.4	33.5	18.1
Occupation*							
Professional/Technical/Manager	5.8	6.8	2.3	6.2	9.2	8.7	13.8
Clerical	1.1	1.8	0.7	0.5	1.5	2.1	2.0
Sales	39.5	34.3	50.5	77.4	45.2	44.2	54.5
Services	6.4	8.3	4.5	6.4	10.7	10.7	11.2
Skilled manual	3.3	3.3	5.5	3.7	3.2	2.9	7.2
Unskilled manual/Agric	19.8	45.3	36.5	5.7	30.1	31.4	11.3
Gender Relations*							
Feminine	5.7	8.3	1.8	3.1	10.4	12.8	12.6
Masculine	62.5	79.3	84.2	92.6	49.8	61.4	65.1
Equal	13.5	12.5	14.0	4.3	39.7	25.8	22.3
ANC Utilization*							
4+	42.1	58.2	44.7	41.2	84.7	68.2	89.0
Less than 4	30.5	41.8	55.3	58.8	15.3	31.8	11.0
Delivery Place*							
Health Facility	30.0	51.5	25.9	15.7	79.8	45.9	76.5
Home / Others	42.6	48.5	74.1	84.3	20.2	54.1	23.5

* Contains missing samples, N (Number of Samples), NC (North-Central), NE (North-East), NW (North-West), SE (South-East), SS (South-South), SW (South-West) regions

### Sociodemographic and spatial dimensions of ANC utilization

The prevalence of RMANC was higher among the 30-34 age cohort except in the NW and SE. The SE also recorded the lowest prevalence among its urban residence compared to other regions. Low education and poor wealth status and lack of media access were also associated with low utilization. It was however higher among respondents with 1-4 children. As further shown in Table 2, women who reported masculine gender relations utilized less ANC services in the north while the pattern was almost evenly distributed among the three gender relations categories in the SE and SW regions. The SW with 89.0% utilization rate had the highest RMANC prevalence in Nigeria followed by the SE (84.7%), SS (68.2%), NC (58.2%), NE (44.7%) and NW (41.2%). The association between gender relations and RMANC was however not statistically significant (P > 0.05) in both the SE and SW regions.

**Table 2:** dimensions of RMANC at national and regional levels using Chi-Square test

Spatial Unit	National (%)	NC (%)	NE (%)	NW (%)	SE (%)	SS (%)	SW (%)
**Variables**							
**Age**							
15-19	44.8	49.7	37.8	37.3	77.2	47.5	84.0
20-24	53.7	55.2	42.3	43.8	85.8	57.8	89.2
25-29	58.4	58.3	45.5	40.6	84.4	70.7	88.9
30-34	62.9	60.5	49.2	42.2	86.1	74.8	90.2
35-39	61.2	60.7	46.7	40.6	82.8	69.3	88.6
40-44	57.7	62.2	42.2	39.1	87.2	71.4	86.7
45-49	54.1	48.0	40.3	39.8	85.4	70.0	91.0
**Marital Status**							
Never in Union	61.5	62.7	41.8	38.5	77.7	52.5	82.3
In Union/Living with a man	57.6	58.0	44.3	41.0	85.6	70.1	89.2
Formerly in Union	64.6	59.7	57.1	50.7	76.1	64.5	92.1
**Types of Residence**							
Urban	75.6	72.8	57.8	59.6	82.9	80.5	91.8
Rural	48.4	51.6	41.3	35.3	88.1	61.8	82.6
**Religion**							
Catholic	76.0	58.7	51.0	65.2	85.2	82.6	85.5
Other Christian	74.7	64.9	62.9	73.7	84.4	67.6	88.9
Islam	46.3	53.5	40.8	40.1	60.0	81.6	89.6
Traditional/Others	40.3	28.6	-	23.1	83.3	42.9	40.0
**Highest Education**							
No education	35.2	35.4	35.2	32.9	58.2	42.4	63.6
Primary	64.2	61.7	55.0	55.5	77.4	57.5	87.0
Secondary	77.2	70.2	68.1	68.3	86.7	68.6	91.7
Higher	91.9	91.1	76.6	87.9	93.2	95.1	96.0
**Partner Highest Education**							
No education	31.0	31.5	29.9	29.0	58.5	34.4	67.6
Primary	61.9	57.1	50.9	47.5	80.2	57.4	85.7
Secondary	72.6	66.0	62.0	57.0	88.3	69.3	91.0
Higher	81.8	80.5	68.9	72.0	92.0	89.7	95.5
**Wealth Status**							
Poor	38.5	39.3	37.8	31.4	68.9	54.7	73.4
Middle	63.6	61.1	55.7	51.9	85.7	58.2	88.0
Rich	81.0	78.3	64.1	69.4	88.7	77.5	92.3
**Children ever born**							
1 - 4	62.6	61.7	46.1	43.6	85.8	70.0	90.8
5 - 8	52.3	51.2	43.2	39.9	83.2	64.2	82.3
9+	40.6	47.4	40.9	35.5	73.3	51.0	81.0
**Experienced Partner Violence**							
Yes	62.1	53.6	46.4	48.0	83.0	71.9	89.1
No	57.4	59.0	44.4	40.7	85.1	67.5	89.0
**Access to Radio/TV**							
Yes	59.8	58.8	46.0	42.2	85.2	72.2	89.0
No	53.7	55.9	38.6	40.0	84.1	60.4	88.8
**Occupation**							
Professional/Technical/Manager	82.9	87.3	67.9	58.3	89.9	87.7	92.0
Clerical	88.5	91.2	76.5	64.7	90.0	100.0	92.9
Sales	62.5	64.8	50.1	44.7	88.1	73.9	90.7
Services	74.2	75.6	48.1	55.2	83.1	74.6	92.1
Skilled manual	73.7	82.1	56.5	49.0	87.8	90.0	92.9
Unskilled manual/Agric	51.8	44.9	46.3	40.5	75.6	52.9	71.6
**Gender Relations**							
Feminine	73.4	66.5	55.0	57.4	84.6	72.3	88.2
Masculine	56.3	57.7	45.1	42.2	85.2	73.8	89.5
Equal	74.2	69.3	56.2	61.1	85.9	66.5	88.5

RMANC (Recommended minimum ANC visits of four), NC (North-Central), NE (North-East), NW (North-West), SE (South-East), SS (South-South), SW (South-West) regions

### Sociodemographic and spatial dimensions of hospital delivery

Like with ANC services utilization, more urban residents used health facilities for delivery except in the South-East. The results also show that hospital delivery was more prevalent among never-married women at the national level and in the NC and NE regions. It was however lowest among adherents of traditional and Islamic religions, and respondents with no education, from poor households, who lacked media access and engaged in unskilled employment / agriculture. The South-East region led in the prevalence of hospital delivery (79.8%) followed by the SW (76.5%), NC (51.5%), SS (45.9%), NE (25.9%) and NW (15.7%). As shown in Table 3, women who reported masculine gender relations had the lowest uptake of hospital delivery except in the South-West region. At both the national level and in the northern regions, the associations between gender relations and hospital delivery were statistically significant (P < 0.05).

**Table 3:** dimensions of health facility delivery at national and regional levels using Chi-Square test

Spatial Unit	National (%)	NC (%)	NE (%)	NW (%)	SE (%)	SS (%)	SW (%)
**Variables**							
**Age**							
15-19	30.1	50.8	24.4	13.5	77.2	26.3	78.0
20-24	36.0	53.1	26.3	15.8	75.7	34.6	73.0
25-29	41.6	49.7	27.2	15.4	81.2	44.8	75.8
30-34	45.9	54.0	25.7	17.1	80.2	53.0	77.9
35-39	45.8	51.2	26.7	16.3	78.6	50.2	79.1
40-44	40.9	53.1	23.8	13.6	84.7	53.0	72.5
45-49	36.9	39.0	20.8	16.3	79.3	46.0	80.8
**Marital Status**							
Never in Union	52.1	62.7	42.9	23.1	71.1	36.1	66.7
In Union/Living with a man	40.7	51.2	25.2	15.4	80.7	47.4	77.0
Formerly in Union	48.3	53.5	35.3	26.3	73.1	37.6	74.2
**Types of Residence**							
Urban	61.7	63.4	48.7	32.4	77.9	62.2	78.0
Rural	30.2	46.2	20.2	10.3	83.4	37.4	73.0
**Religion**							
Catholic	70.3	61.3	25.5	45.5	79.6	66.7	77.1
Other Christian	61.0	59.8	44.1	34.9	80.1	43.5	77.0
Islam	26.2	43.2	22.2	14.8	60.0	83.7	75.8
Traditional/Others	27.3	35.7	-	5.1	66.7	27.6	60.0
**Highest Education**							
No education	15.3	27.6	15.6	8.7	43.0	22.7	56.0
Primary	42.5	52.2	31.2	20.5	59.8	29.6	69.9
Secondary	64.1	65.5	54.4	40.1	84.8	45.1	77.4
Higher	87.2	87.6	72.4	76.9	96.1	85.7	90.3
**Partner Highest Education**							
No education	12.7	23.1	12.3	6.6	26.8	31.3	62.6
Primary	40.7	45.5	23.5	13.1	70.5	29.3	69.7
Secondary	56.6	59.9	40.9	25.8	86.1	43.4	76.5
Higher	70.8	78.6	56.2	6.6	92.4	78.3	87.0
**Wealth Status**							
Poor	18.8	33.7	17.2	7.3	46.7	26.1	63.3
Middle	44.5	54.1	33.9	20.4	75.7	33.7	74.2
Rich	70.0	70.6	59.0	44.9	91.1	58.3	79.7
**Children ever born**							
1 - 4	47.4	56.1	28.7	17.5	82.6	48.6	78.5
5 - 8	33.9	43.2	22.8	14.4	75.3	38.8	69.7
9+	18.6	28.9	20.5	11.9	55.0	32.7	52.4
**Experienced Partner Violence**							
Yes	46.4	52.0	28.1	17.9	78.8	42.7	72.5
No	40.6	51.5	25.6	15.5	80.0	46.6	77.0
**Access to Radio/TV**							
Yes	43.2	51.8	27.0	15.5	80.0	50.3	77.1
No	36.9	50.2	22.1	15.9	79.5	37.3	74.8
**Occupation**							
Professional/Technical/Manager	71.9	82.5	47.2	34.1	85.9	76.1	83.3
Clerical	86.1	94.1	82.4	76.5	90.0	80.0	85.7
Sales	42.9	56.6	28.8	15.3	86.3	48.1	77.2
Services	59.4	72.8	28.7	26.4	80.8	50.7	75.9
Skilled manual	58.5	70.5	40.1	28.5	86.5	63.3	79.3
Unskilled manual/Agric	36.9	41.1	21.5	13.5	58.2	33.3	60.3
**Gender Relations**							
Feminine	57.8	63.7	26.7	24.1	80.9	50.5	71.4
Masculine	38.1	50.5	26.0	15.8	79.0	49.4	77.3
Equal	61.2	66.6	31.3	28.4	82.7	45.6	78.4

NC (North-Central), NE (North-East), NW (North-West), SE (South-East), SS (South-South), SW (South-West) regions

### Determinants of RMANC

At the national level, the regression models show that age, education, wealth status, and masculine gender relations increase the likelihood of achieving the RMANC while place of residence, religion, number of children, experience of intimate partner violence, media access, occupation and feminine gender relations were associated with lower odds of utilization. The regional patterns of association of the variables reflect the national pattern and are similar in all the regions with respect to education, wealth, number of children, intimate partner violence, media access and occupation. At the unadjusted level, masculine gender relations were found to significantly increase the odds of achieving RMANC at the national level and the three northern regions (Table 4). In the South-South region, masculine gender relations lowered the odds. Feminine gender relations were not statistically significant in their relationships with RMANC in any of the spatial units. In the adjusted models and controlling for the sociodemographic confounders (Table 5), gender relations (masculine) were only significant in the North-Central and South-South regions. They increased the odds in the former but were associated with lower odds in the latter.

**Table 4:** results of the multivariate logistics regression of the relationship between gender relations and RMANC

Spatial Unit	National			North-Central			North-East			North-West			South-East			South-South			South-West		
	OR	95% CI		OR	95% CI		OR	95% CI		OR	95% CI		OR	95% CI		OR	95% CI		OR	95% CI	
Gender Relations		LL	UL		LL	UL		LL	UL		LL	UL		LL	UL		LL	UL		LL	UL
Feminine	1.04	0.88	1.23	1.14	0.79	1.62	1.05	0.61	1.80	1.16	0.75	1.79	1.11	0.69	1.78	0.76	0.52	1.10	1.03	0.63	1.69
Masculine	2.24*	2.04	2.44	1.66*	1.32	2.08	1.56*	1.29	1.89	2.14*	1.61	2.85	1.06	0.82	1.38	0.70*	0.56	0.89	0.90	0.66	1.24
Equal	RC			RC			RC			RC			RC			RC			RC		

* Significant at P < 0.05, OR: Odds Ratio, CI: Confidence Interval, LL: Lower CI Limit, UL: Upper CI Limit, RC: Reference Category, RMANC (Recommended minimum ANC visits of four)

**Table 5:** results of the controlled multivariate logistics regression of the relationship between gender relations and RMANC

Spatial Unit	National			North-Central			North-East			North-West			South-East			South-South			South-West		
	OR	95% CI		OR	95% CI		OR	95% CI		OR	95% CI		OR	95% CI		OR	95% CI		OR	95% CI	
Age		LL	UL		LL	UL		LL	UL		LL	UL		LL	UL		LL	UL		LL	UL
15-19	1.51*	1.10	2.07	1.35	0.63	2.89	1.32	0.69	2.53	0.75	0.43	1.30	5.20*	1.48	8.27	3.84*	1.13	13.00	5.55*	1.18	16.05
20-24	1.44*	1.12	1.86	1.19	0.62	2.29	1.02	0.59	1.75	1.04	0.66	1.64	1.73	0.62	4.83	3.10*	1.28	7.55	3.10*	1.08	8.96
25-29	1.34*	1.06	1.71	1.02	0.54	1.90	1.01	0.61	1.68	1.13	0.74	1.72	2.54*	1.01	6.42	2.28	0.97	5.32	2.93*	1.08	7.95
30-34	1.12	0.89	1.41	0.99	0.53	1.83	0.72	0.44	1.18	1.15	0.77	1.71	1.94	0.78	4.81	1.43	0.62	3.26	2.55	0.96	6.80
35-39	1.08	0.86	1.36	0.65	0.35	1.20	0.82	0.51	1.31	1.09	0.74	1.61	2.32	0.94	5.71	1.52	0.68	3.39	2.59	0.99	6.75
40-44	0.90	0.71	1.14	0.68	0.36	1.28	0.81	0.50	1.32	0.96	0.65	1.42	1.34	0.51	3.49	0.98	0.42	2.32	1.68	0.61	4.65
45-49	RC			RC			RC			RC			RC			RC			RC		
**Residence**																					
Urban	0.85*	0.77	0.95	0.65*	0.51	0.84	0.96	0.73	1.27	0.83	0.69	1.01	1.55*	1.14	2.11	0.58*	0.42	0.81	0.91	0.62	1.33
Rural	RC			RC			RC			RC			RC			RC			RC		
**Religion**																					
Catholic	0.50*	0.29	0.86	0.24	0.05	1.20	0.85	0.62	0.97	0.39	0.12	1.29	4.42	0.51	8.02	0.39	0.14	1.10	0.07	0.00	1.05
Other Xtian	0.55*	0.33	0.92	0.27	0.05	1.37	1.45	1.14	1.62	0.27*	0.10	0.76	4.89	0.57	9.93	0.73	0.29	1.88	0.06*	0.00	0.93
Islam	0.65	0.39	1.10	0.29	0.06	1.46	1.11	0.92	1.33	0.84	0.32	2.24	6.57	0.62	11.32	0.21*	0.05	0.91	0.05*	0.00	0.67
Traditional	RC			RC			RC			RC			RC			RC			RC		
**High Edu**																					
None	3.72*	2.84	4.87	3.87*	2.17	6.89	1.57	0.83	2.99	5.70*	2.70	9.03	3.29*	1.32	8.20	7.27*	2.81	18.82	5.71*	2.61	12.49
Primary	2.39*	1.83	3.11	2.43*	1.37	4.28	1.09	0.57	2.09	3.72*	1.74	7.93	1.84	0.92	3.70	3.43*	1.49	7.89	2.08	0.98	4.44
Secondary	1.87*	1.46	2.39	2.02*	1.18	3.45	0.95	0.51	1.77	3.33*	1.59	6.99	1.31	0.70	2.43	2.52*	1.16	5.50	1.60	0.84	3.05
Higher	RC			RC			RC			RC			RC			RC			RC		
**P.Education**																					
None	2.25*	1.91	2.66	2.09*	1.42	3.07	2.74*	1.95	3.85	2.05*	1.53	2.75	2.19	0.81	5.90	2.84*	1.13	7.11	2.76*	1.40	5.42
Primary	1.34*	1.13	1.60	1.21	0.83	1.77	1.74*	1.20	2.52	1.15	0.83	1.59	1.61	0.83	3.12	1.76	0.96	3.22	1.21	0.63	2.35
Secondary	1.24*	1.07	1.44	1.07	0.78	1.48	1.28	0.92	1.77	1.35*	1.01	1.80	1.18	0.63	2.23	2.18*	1.29	3.66	1.37	0.81	2.32
Higher	RC			RC			RC			RC			RC			RC			RC		
**Wealth**																					
Poor	1.89*	1.66	2.16	2.32*	1.74	3.10	1.55*	1.10	2.20	1.91*	1.45	2.52	2.21*	1.44	3.37	1.53*	1.05	2.22	1.71*	1.06	2.76
Middle	1.35*	1.19	1.53	1.44*	1.09	1.90	1.28	0.91	1.79	1.27	0.97	1.66	1.15	0.81	1.65	1.36	0.98	1.90	0.89	0.57	1.40
Rich	RC			RC			RC			RC			RC			RC			RC		
**CEB**																					
1 - 4	0.64*	0.53	0.78	0.84	0.48	1.49	0.86	0.59	1.25	0.84	0.60	1.16	0.70	0.31	1.60	0.43*	0.19	0.94	0.33	0.09	1.24
5 - 8	0.75*	0.63	0.88	1.14	0.67	1.95	0.96	0.71	1.31	0.69*	0.54	0.90	0.65	0.29	1.44	0.60	0.28	1.27	0.60	0.16	2.16
9+	RC			RC			RC			RC			RC			RC			RC		
**Violence**																					
Yes	0.95	0.84	1.07	1.17	0.92	1.49	0.92	0.73	1.17	0.76	0.56	1.02	1.08	0.77	1.52	0.84	0.59	1.18	1.00	0.60	1.65
No	RC			RC			RC			RC			RC			RC			RC		
**Radio/TV**																					
Yes	0.85*	0.78	0.93	0.93	0.76	1.15	0.82	0.66	1.02	0.90	0.77	1.04	1.03	0.77	1.39	0.67*	0.51	0.89	0.97	0.64	1.47
No	RC			RC			RC			RC			RC			RC			RC		
**Occupaton**																					
Pro / Mgr	0.76*	0.61	0.95	0.69	0.40	1.19	1.24	0.58	2.62	0.69	0.40	1.19	0.75	0.39	1.43	0.44*	0.22	0.87	0.89	0.47	1.69
Clerical	0.68	0.39	1.19	0.20	0.03	1.53	0.69	0.17	2.77	2.29	0.63	8.25	0.97	0.27	3.49	0.14	0.00		1.19	0.32	4.39
Sales	0.76*	0.68	0.85	0.83	0.66	1.05	0.90	0.73	1.12	0.65	0.42	1.02	0.84	0.57	1.22	0.54*	0.40	0.74	0.61*	0.39	0.97
Services	0.74*	0.62	0.89	0.55*	0.37	0.81	1.33	0.84	2.11	0.65	0.39	1.09	1.27	0.79	2.07	0.51*	0.32	0.81	0.61	0.32	1.15
Skilled	0.58*	0.47	0.72	0.41*	0.23	0.73	0.77	0.53	1.13	0.61	0.35	1.07	0.63	0.25	1.58	0.12*	0.04	0.41	0.49	0.23	1.05
Unskilled	RC			RC			RC			RC			RC			RC			RC		
**G. Relations**																					
Feminine	0.93	0.76	1.14	1.31	0.85	2.03	1.01	0.54	1.87	0.85	0.48	1.51	1.05	0.63	1.74	0.70	0.45	1.10	1.44	0.82	2.55
Masculine	1.04	0.92	1.17	1.42*	1.06	1.90	0.94	0.74	1.20	1.49	0.99	2.22	0.94	0.70	1.26	0.68*	0.51	0.93	0.93	0.64	1.34
Equal	RC			RC			RC			RC			RC			RC			RC		

* Significant at P < 0.05, OR: Odds Ratio, CI: Confidence Interval, LL: Lower CI Limit, UL: Upper CI Limit, RC: Reference Category, RMANC (Recommended minimum ANC visits of four), High Edu: Highest Education, P.Education: Partner’s Education, CEB: Children Ever Born, G.Relations: Gender Relations

### Determinants of health facility for delivery

Feminine gender relations were not statistically significant in their relationship with hospital delivery at the national level and in all the regions except in the South-West in the unadjusted models (Table 6). While masculine gender relations were associated with increased likelihoods of hospital delivery at the national level and in the NC, NE, NW and SE, they showed no significant relationships with hospital delivery in the SS and SW regions. The introduction of the sociodemographic confounders in the adjusted models changed the relationships at the national level and most of the regions. Neither feminine nor masculine gender relations significantly predicted hospital delivery at the national level and in the NE, NW, SE and SS. Masculine relations increased the odds of hospital delivery in the North-Central, while feminine relations increased the odds in the South-West region (Table 7). While age was a significant predictor of hospital delivery at the national level, it was not significant in the northern regions at the subnational level. The same was place of residence was not significant at the national level, but lowers utilization in the NW and SS and increase the likelihoods in the SE. Education and wealth status increase utilization at national and subnational levels, while number of children were associated with lower odds. Religion was only significant in lowering the odds in the South-South region.

**Table 6:** results of the multivariate logistics regression of the relationship between gender relations and hospital delivery

Spatial Unit	National			North-Central			North-East			North-West			South-East			South-South			South-West		
	OR	95% CI		OR	95% CI		OR	95% CI		OR	95% CI		OR	95% CI		OR	95% CI		OR	95% CI	
Gender Relations		LL	UL		LL	UL		LL	UL		LL	UL		LL	UL		LL	UL		LL	UL
Feminine	1.15	0.99	1.34	1.14	0.80	1.61	1.26	0.69	2.29	1.25	0.76	2.03	1.13	0.74	1.74	0.82	0.58	1.15	1.46*	1.02	2.09
Masculine	2.57*	2.37	2.79	1.96*	1.57	2.44	1.30*	1.06	1.60	2.11*	1.55	2.88	1.27*	1.01	1.61	0.86	0.69	1.07	1.06	0.84	1.35
Equal	RC			RC			RC			RC			RC			RC			RC		

* Significant at P < 0.05, OR: Odds Ratio, CI: Confidence Interval, LL: Lower CI Limit, UL: Upper CI Limit, RC: Reference Category

**Table 7:** results of the controlled multivariate logistics regression of the relationship between gender relations and hospital delivery

Spatial Unit	National			North-Central			North-East			North-West			South-East			South-South			South-West		
	OR	95% CI		OR	95% CI		OR	95% CI		OR	95% CI		OR	95% CI		OR	95% CI		OR	95% CI	
Age		LL	UL		LL	UL		LL	UL		LL	UL		LL	UL		LL	UL		LL	UL
15-19	1.66*	1.17	2.35	0.77	0.35	1.68	1.10	0.50	2.46	1.06	0.51	2.22	5.38*	1.43	20.28	4.71*	1.38	16.08	2.68	0.75	9.57
20-24	1.82*	1.38	2.40	0.84	0.43	1.63	0.97	0.50	1.90	1.18	0.64	2.17	5.82*	2.17	15.59	4.48*	1.88	10.70	3.28*	1.49	7.21
25-29	1.90*	1.46	2.47	1.04	0.55	1.97	1.20	0.64	2.27	1.53	0.87	2.70	4.29*	1.71	10.79	3.62*	1.58	8.27	2.93*	1.38	6.23
30-34	1.55*	1.20	2.00	0.94	0.50	1.78	0.93	0.51	1.72	1.42	0.84	2.42	4.21*	1.71	10.36	1.96	0.88	4.38	2.65*	1.26	5.56
35-39	1.34*	1.04	1.72	0.98	0.52	1.83	0.79	0.44	1.43	1.25	0.74	2.09	3.79*	1.55	9.29	1.45	0.66	3.17	2.19*	1.05	4.57
40-44	1.19	0.91	1.54	0.83	0.43	1.59	0.92	0.50	1.68	1.39	0.81	2.39	1.73	0.68	4.42	1.00	0.44	2.29	2.44*	1.14	5.22
45-49	RC			RC			RC			RC			RC			RC			RC		
**Residence**																					
Urban	0.95	0.85	1.05	0.82	0.64	1.05	0.78	0.58	1.05	0.77*	0.60	0.98	1.49*	1.12	2.00	0.50*	0.38	0.66	1.22	0.93	1.60
Rural	RC			RC			RC			RC			RC			RC			RC		
**Religion**																					
Catholic	0.74	0.42	1.33	1.04	0.28	3.95	0.85	0.62	0.95	0.36	0.07	1.87	1.42	0.33	6.17	0.59	0.21	1.64	0.70	0.06	8.95
Other Xtian	1.05	0.60	1.85	1.56	0.42	5.84	1.02	0.86	1.38	0.89	0.19	4.20	1.47	0.34	6.38	1.27	0.49	3.29	0.81	0.07	9.58
Islam	1.31	0.74	2.32	2.30	0.62	8.56	0.95	0.70	1.26	1.56	0.34	7.23	0.45	0.11	0.52	0.08*	0.02	0.38	0.69	0.06	8.24
Traditional	RC			RC			RC			RC			RC			RC			RC		
**High Edu**																					
None	5.05*	3.98	6.42	3.77*	2.23	6.38	3.13*	1.62	6.01	5.39*	3.08	9.45	9.48*	3.33	27.06	13.83*	5.78	33.10	4.47*	2.53	7.90
Primary	3.36*	2.67	4.23	2.35*	1.41	3.94	2.09*	1.09	4.04	3.49*	1.97	6.17	8.19*	3.46	19.35	4.96*	2.63	9.33	2.81*	1.70	4.62
Secondary	2.30*	1.87	2.84	2.07*	1.28	3.35	1.27	0.68	2.37	2.89*	1.71	4.90	3.82*	1.69	8.61	2.94*	1.70	5.09	2.16*	1.43	3.27
Higher	RC			RC			RC			RC			RC			RC			RC		
**P.Education**																					
None	2.53*	2.13	3.00	2.69*	1.84	3.94	2.78*	1.94	3.97	2.69*	1.93	3.76	3.26*	1.17	9.05	1.10	0.44	2.76	1.49	0.91	2.46
Primary	1.73*	1.47	2.05	1.79*	1.24	2.59	1.84*	1.24	2.72	1.79*	1.23	2.63	1.26	0.66	2.41	2.23*	1.34	3.71	1.36	0.88	2.10
Secondary	1.36*	1.18	1.56	1.43*	1.06	1.94	1.13	0.82	1.56	1.47*	1.09	1.98	0.98	0.52	1.83		1.52	3.35	1.39	1.00	1.95
Higher	RC			RC			RC			RC			RC			RC			RC		
**Wealth**																					
Poor	2.39*	2.10	2.73	2.25*	1.70	2.99	2.922*	2.03	4.21	2.39*	1.72	3.32	5.61*	3.78	8.34	1.93*	1.31	2.84	1.44*	1.01	2.07
Middle	1.46*	1.30	1.64	1.33*	1.02	1.73	2.01*	1.43	2.83	1.33	0.99	1.78	2.56*	1.84	3.58	1.45*	1.06	1.99	0.91	0.67	1.24
Rich	RC			RC			RC			RC			RC			RC			RC		
**CEB**																					
1 - 4	0.57*	0.46	0.71	0.60	0.33	1.09	1.04	0.66	1.65	0.81	0.51	1.28	0.27*	0.12	0.61	0.39*	0.17	0.92	0.23*	0.08	0.67
5 - 8	0.72*	0.59	0.88	0.69	0.39	1.23	1.10	0.75	1.61	0.79	0.55	1.13	0.33*	0.15	0.72	0.70	0.31	1.58	0.32*	0.11	0.94
9+	RC			RC			RC			RC			RC			RC			RC		
**Violence**																					
Yes	1.02	0.90	1.15	0.88	0.69	1.12	0.93	0.70	1.23	0.99	0.67	1.47	0.87	0.62	1.22	1.14	0.83	1.57	1.40*	1.01	1.95
No	RC			RC			RC			RC			RC			RC			RC		
**Radio/TV**																					
Yes	0.94	0.86	1.04	1.01	0.82	1.24	0.82	0.62	1.08	1.15	0.94	1.41	1.25	0.94	1.66	0.80	0.62	1.04	0.81	0.62	1.07
No	RC			RC			RC			RC			RC			RC			RC		
**Occupation**																					
Pro / Mgr	0.89	0.72	1.10	0.74	0.45	1.22	2.45*	1.11	5.43	0.63	0.31	1.28	1.17	0.65	2.11	0.63	0.36	1.12	0.74	0.46	1.21
Clerical	0.49*	0.29	0.82	0.29	0.07	1.30	0.19	0.03	1.05	0.26	0.06	1.10	1.03	0.27	3.92	0.33	0.09	1.16	0.91	0.34	2.43
Sales	0.79*	0.70	0.89	0.83	0.65	1.05	0.70*	0.53	0.92	0.68	0.37	1.24	0.64*	0.45	0.91	0.87	0.63	1.19	0.74	0.51	1.08
Services	0.81*	0.68	0.96	0.55*	0.38	0.80	1.16	0.65	2.05	0.63	0.32	1.24	1.10	0.69	1.74	0.85	0.55	1.31	0.77	0.48	1.22
Skilled	0.55*	0.45	0.68	0.63	0.38	1.04	0.51*	0.33	0.79	0.29*	0.15	0.61	0.77	0.35	1.74	0.46*	0.22	0.94	0.66	0.39	1.11
Unskilled	RC			RC			RC			RC			RC			RC			RC		
**G.Relations**																					
Feminine	1.04	0.86	1.26	1.27	0.83	1.94	1.16	0.55	2.45	0.65	0.34	1.28	0.91	0.55	1.50	0.70	0.45	1.07	1.59*	1.07	2.36
Masculine	1.06	0.94	1.19	1.50*	1.13	2.00	0.85	0.64	1.12	0.99	0.61	1.61	1.05	0.78	1.39	0.75	0.56	1.00	1.03	0.79	1.33
Equal	RC			RC			RC			RC			RC			RC			RC		

* Significant at P < 0.05, OR: Odds Ratio, CI: Confidence Interval, LL: Lower CI Limit, UL: Upper CI Limit, RC: Reference Category, High Edu: Highest Education, P.Education: Partner’s Education, CEB: Children Ever Born, G.Relations: Gender Relations

## Discussion

To summarize the findings, only 5.7% of the women exhibited feminine gender relations at the national level. The percentages were however higher than the national average in the North-Central (8.3%), South-East (10.4%), South-South (12.8%) and South-West (12.6%) regions. Masculine gender relations were dominant in the population. RMANC and health facility delivery were also higher than the national average of 42.1% and 30.0% respectively in all the regions except the North-East and North-West. The impacts of gender relations on the utilization of the selected maternal healthcare services varied between the national and subnational, and within the regions at the subnational level. While feminine gender relations were not statistically significant in their relationships with RMANC at both national and subnational levels, masculine gender relations were associated with increased likelihoods that women would use health facilities for delivery at the national level and in the NC, NE, NW and SE regions.

The first indication that gender relations would have an unequal impact among different ethnic groups in their regional clusters was in the spatial distribution of place of residence, religious affiliation and education in the regions. Urban residency, Christianity of other denominations other than Catholic and higher education are associated with some flexibility that may impact the acceptance of women autonomy in the households. From the findings, there were more urban residents in the SE and SW, Islam was more prevalent in the northern regions, and respondents with at least secondary education and whose spouses had at least secondary education were more prevalent in the southern regions. Consequently, while the national prevalence of feminine gender relations was 5.7%, the prevalence was below the national average in the largely Islamic, more rural and less educated NE and NW. Although relatively low in the southern regions as well, the prevalence was much higher than the national average. RMANC in the northern regions was also lower than in the South while the uptake of health facility delivery was lower than the national average of 30.0% in the NE and NW. These findings further confirm that place, religion and education affect maternal health seeking [7,10,21,22], and that there is a spatial variation in health outcomes in different regions of Nigeria based on the sociodemographic composition of the regions [13, 37-40].

It was therefore not surprising that at the level of regression to determine predictive relationship, masculine gender relations predicted increased odds of RMANC and health facility delivery at the national level and in all the regions except the SS and SW while feminine gender relations were associated with increased likelihood of using health facilities for delivery only in the SW region. The findings show that women autonomy on its own does not predict utilization of maternal healthcare services, otherwise, feminine gender relations would have been a significant predictor of utilization in the SS and SE regions with the highest prevalence of never married and previously married mothers. This departs from findings in previous studies that women autonomy predicts increased usage of maternal healthcare services in Nigeria [10, 11, 34-36]. Our findings rather confirm that women who take part in decision-making were less likely to use maternal healthcare services when compared with their counterparts with limited autonomy [23]. In the SW where women autonomy was significant, the women were more urbanized, more educated, richer, had higher media access, were less Catholic than women in other southern regions, and practised Islam less than the women in the predominantly Islamic north.

## Conclusion

Gender relations in Nigeria are unequal in how they are expressed across different subnational levels and as such, their impacts on the uptake of maternal healthcare services are not uniform. While feministic gender relations were, understandably, more prevalent among never married and previously married mothers in the study area, they were substantially low among married mothers and exerted no statistically significant influence on the uptake of maternal healthcare services except in the SW region. The peculiarity of the region with respect to education, religion, media exposure and wealth status, rather than the feminine gender relations themselves, accounted for the exception in the SW region. Since women with more autonomy were found to be less likely to use maternal healthcare services, deemphasizing union formation to allow for improved women autonomy as being canvassed by local feminist movements, apart from its documented negative impacts on children´s health and development, may also not positively improve utilization. Rather, it is recommended that men, especially in the NE and NW regions where uptake is lowest, be educated on the benefits of utilization so they can support their spouses.

### What is known about this topic

- Maternal health outcomes in Nigeria are among the poorest in the world;
- Low level of utilization of maternal healthcare services contributes to the poor outcomes.

### What this study adds

- Gender relations within the household possess the capacity to improve the utilization of maternal healthcare services;
- Levels of gender relations are not uniform across subnational regions in Nigeria and have varying effects across the regions;
- On its own, feminine gender relations do not increase the uptake of maternal healthcare services.
